# Canine meningiomas are comprised of 3 DNA methylation groups that resemble the molecular characteristics of human meningiomas

**DOI:** 10.1007/s00401-024-02693-2

**Published:** 2024-02-20

**Authors:** Naomi Zakimi, Christina N. Mazcko, Christine Toedebusch, Gregory Tawa, Kevin Woolard, Amy K. LeBlanc, Peter J. Dickinson, David R. Raleigh

**Affiliations:** 1 Department of Radiation Oncology, University of California San Francisco, San Francisco, CA USA; 2 Department of Neurological Surgery, University of California San Francisco, San Francisco, CA USA; 3 Department of Pathology, University of California San Francisco, San Francisco, CA USA; 4 Comparative Oncology Program, Center for Cancer Research, National Cancer Institute, National Institutes of Health, Bethesda, MD USA; 5 Department of Surgical and Radiological Sciences, School of Veterinary Medicine, University of California Davis, Davis, CA USA; 6 Therapeutic Development Branch, Division of Preclinical Innovation, National Center for Advancing Translational Sciences, National Institutes of Health, Bethesda, MD USA; 7 Department of Pathology, Microbiology, and Immunology, School of Veterinary Medicine, University of California Davis, Davis, CA USA

Meningiomas are the most common primary intracranial tumor in humans [16] and in dogs [18, 20]. Unsupervised analyses of DNA methylation or RNA sequencing data from human meningiomas reveal three molecular groups of tumors that are associated with distinct biological drivers, therapeutic vulnerabilities, and clinical outcomes [5, 17]. These consensus molecular groups are concordant with results of integrated clustering analyses of copy number variants (CNVs), DNA methylation profiles, RNA sequencing data, and somatic short variants, which suggest that molecular groups of human meningiomas can be comprised of subgroups [4, 15]. Here we test the hypothesis that comparative understanding of the molecular characteristics of canine meningiomas may shed light on the tractability of these naturally occurring tumors as models for meningiomas in humans [9], knowledge that might be particularly useful given the limitations of genetically engineered mouse models of meningioma [6].

Whole genome bisulfide sequencing (WGBS) was performed on 29 canine meningiomas spanning all histological grades, a diversity of intracranial and spinal locations, and 23 breeds of dogs (Supplementary Table 1). Most canine meningiomas had meningothelial or microcystic architecture and were CNS WHO grade 1 (19 of 29 tumors), but there were 5 CNS WHO grade 2 meningiomas and 5 CNS WHO grade 3 meningiomas. CpG methylation sequencing data were processed using three parallel clustering methods to identify molecular groups of canine meningiomas (Methods, online resource), including (1) k-means clustering to partition samples into stable groups based on CpG features, (2) unsupervised hierarchical clustering to identify nested groups based on Euclidean distance metrics, and (3) dimensionality reduction with principle component analysis, uniform manifold approximation and projection (UMAP), and t-distributed Stochastic Neighbor Embedding (t-SNE). Consensus k-means clustering revealed loss of coherence beyond 3 DNA methylation groups (Fig. 1a), and Monte Carlo simulation of within-cluster dispersion in comparison to a null reference distribution also supported the existence of 3 DNA methylation groups of canine meningiomas, similar to human meningiomas (Fig. 1b). Continuous distribution functions (Fig. 1c, d), unsupervised hierarchical clustering (Fig. 1e), and dimensionality reduction (Fig. 1f) were also consistent with 3 DNA methylation groups of canine meningiomas.

**Fig. 1:**
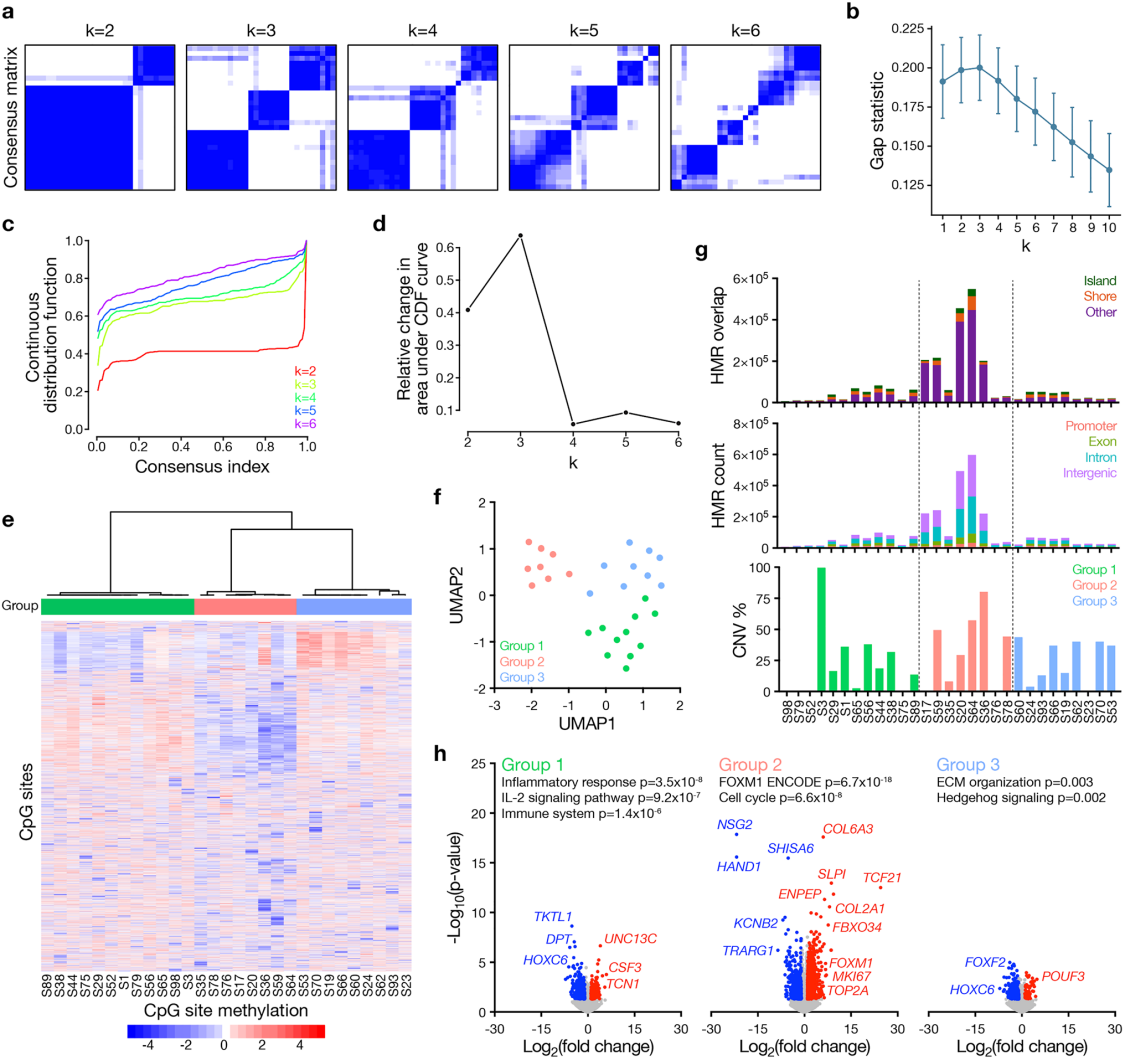
Canine meningiomas are comprised of 3 molecular groups. **a** Consensus matrix with k = 2–6 to evaluate cluster stability from WGBS of 29 canine meningiomas. **b** Gap statistic shows the optimal number of canine meningioma groups from WGBS is 3. **c** Continuous distribution function (CDF) for k-means consensus clustering supports 3 distinct molecular groups of canine meningiomas. **d** Relative change in area under the CDF curve, highlighting k = 3 as the optimal number of canine meningioma groups. **e** Heatmap representing methylation status at all CpG sites across k = 3 groups. **f** Uniform manifold approximation and projection (UMAP) visualization of WGBS data from canine meningiomas using n = 15 neighbors. **g** Sample-Level DNA methylation and genomic feature analysis showing hypomethylated regions (HMR) overlapping with CpG islands or shores, the frequency of HMRs across genomic elements, and the extent of the genome affected by copy number variants (CNV). **h** Volcano plots showing differentially expressed genes from paired RNA sequencing of canine meningiomas according to molecular groups from WGBS. Gene ontologies from ENRICHR are shown for differentially enriched genes in each molecular group. Significantly enriched (red) or suppressed (blue) genes have log_2_ fold-change > 1 and p-value < 0.05

Most genomic CpG sites are methylated, but hypomethylated regions (HMRs) that are comprised of islands, shores (± 2 kb around islands), and shelves (± 2 kb around shores) can be associated with dysregulated gene expression [19]. To elucidate genomic differences across DNA methylation groups of canine meningiomas, HMRs and CNVs were defined from WGBS data. CNVs were distributed across DNA methylation groups, but Group 2 meningiomas were enriched in HMRs (Fig. 1g), suggesting this molecular group may be associated with particularly dysregulated gene expression. To test this hypothesis, RNA sequencing was performed on the same 29 samples to determine if the gene expression programs underlying DNA methylation groups of canine meningiomas resembled the gene expression programs underlying molecular groups of human meningiomas (Supplementary Table 2). In humans, meningiomas from the Merlin-intact DNA methylation group have the best outcomes and can encode somatic short variants targeting the Hedgehog pathway [4, 5, 15, 17]. Gene ontology analysis of RNA sequencing data from Group 3 canine meningiomas revealed enrichment of Hedgehog target genes compared to Group 1 and Group 2 tumors (Fig. 1h). In humans, meningiomas from the Immune-enriched DNA methylation group have intermediate outcomes and are distinguished by tumor immune infiltration, intratumor lymphatic vessels, and focal copy number amplification of the *HLA* locus [5, 17]. Gene ontology analysis of RNA sequencing data from Group 1 canine meningiomas revealed enrichment of inflammatory, immune, and IL-2 signaling pathways in comparison to Group 2 and Group 3 tumors (Fig. 1h). In humans, meningiomas from the Hypermitotic DNA methylation group have the worst outcomes and are distinguished by convergent genetic and epigenetic mechanisms that mis-activate the cell cycle, including enrichment of the FOXM1 gene expression program [5, 17]. Gene ontology analysis of RNA sequencing data from Group 2 canine meningiomas, which were enriched for HMRs (Fig. 1g) and cell proliferation pathways (Supplementary Fig. 1, online resource), revealed enrichment of *FOXM1*, the FOXM1 gene expression program, and cell cycle target genes in comparison to Group 1 and Group 3 tumors (Fig. 1h). In support of these findings, the average histological mitotic index was higher for Group 2 compared to Group 1 and Group 3 tumors (6 versus 1, p = 0.0003, Student’s t test), as were the transcripts per megabase expression of the cell proliferation markers *MKI67* (152 versus 26, p < 0.0001, Student’s t test) and *TOP2A* (1289 versus 255, p < 0.0001, Student’s t test) (Fig. 1h and Supplementary Table 1). Moreover, Group 2 canine meningiomas were more likely to be high-grade (CNS WHO grade 2 or grade 3) compared to Group 1 and Group 3 tumors (p = 0.02, Student’s t test) (Supplementary Table 1). There were no apparent trends in morphological subtypes across molecular groups of canine meningiomas, but robust analysis of morphological subtypes, which phenocopy human meningiomas, was likely impaired by small sample size (Supplementary Table 1 and Supplementary Fig. 2, online resource).

In conclusion, naturally occurring canine meningiomas are comprised of 3 DNA methylation groups that resemble the molecular characteristics of human meningiomas. These data suggest that canine meningioma patients may be suitable models for preclinical pharmacological testing, which may ultimately improve clinical outcomes for bipedal and quadrupedal meningioma patients alike. As with human meningiomas, molecular and systemic therapies remain ineffective or experimental for canine meningiomas [14], and many owners opt for palliative care or euthanasia at the time of a brain tumor diagnosis. Thus, clinical outcome data were not available for the cohort of canine meningiomas reported here, and more samples may ultimately be necessary to determine if molecular groups of canine meningiomas harbor molecular subgroups, as in human meningiomas [4, 15]. In the interim, these data provide a foundation for future investigation of a molecularly tractable, naturally occurring model of the most common primary intracranial tumor in humans and shed light on the evolutionary conservation of meningiomas across higher vertebrates.

### Supplementary Information

Below is the link to the electronic supplementary material.

Supplementary file1 (PDF 4098 KB)

## Data Availability

Whole genome bisulfide sequencing and RNA sequencing data for all canine meningiomas reported and analyzed in this study have been deposited to the NCBI Gene Expression Omnibus under access numbers PRJNA1046409 and PRJNA1045298.
