# Supplementary material for: Canine meningiomas are comprised of 3 DNA methylation groups that resemble the molecular characteristics of human meningiomas
Source: Acta Neuropathol. 2024 Feb 20;147(1):43. doi: 10.1007/s00401-024-02693-2 (PMC10879255; doi:10.1007/s00401-024-02693-2)
Supplement: Supplementary file 1 — Supplementary file1 (PDF 4098 KB) [file 401_2024_2693_MOESM1_ESM.pdf]

ONLINE RESOURCES

Supplementary Fig. 1. Gene expression programs in canine meningiomas. Visualization of pathway enrichment (red) or suppression (blue) from RNA sequencing of protein coding genes in canine meningiomas (n=29). Differentially expressed pathways in each group were iteratively compared to the union of pathways in other groups. Nodes represent pathways and edges represent shared genes between pathways (p≤0.05, FDR≤0.05).

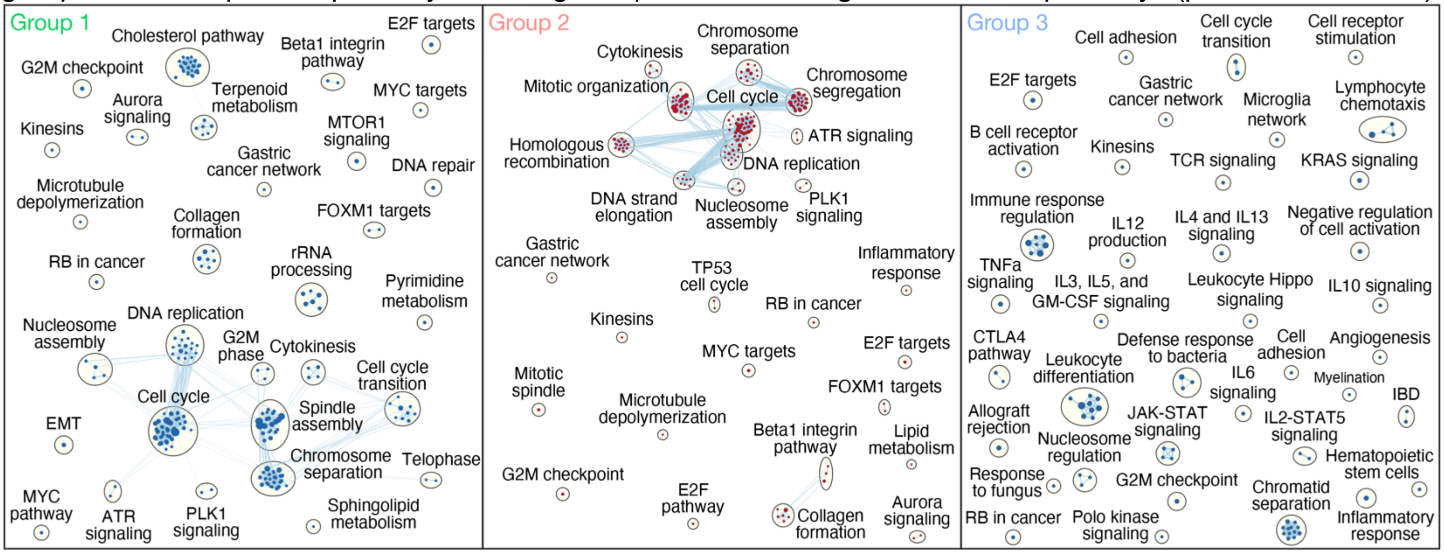

Supplementary Fig. 2. Representative histology across molecular groups of canine meningiomas. Sample number and morphology for each sample are provided. Scale bars, 50 μm.

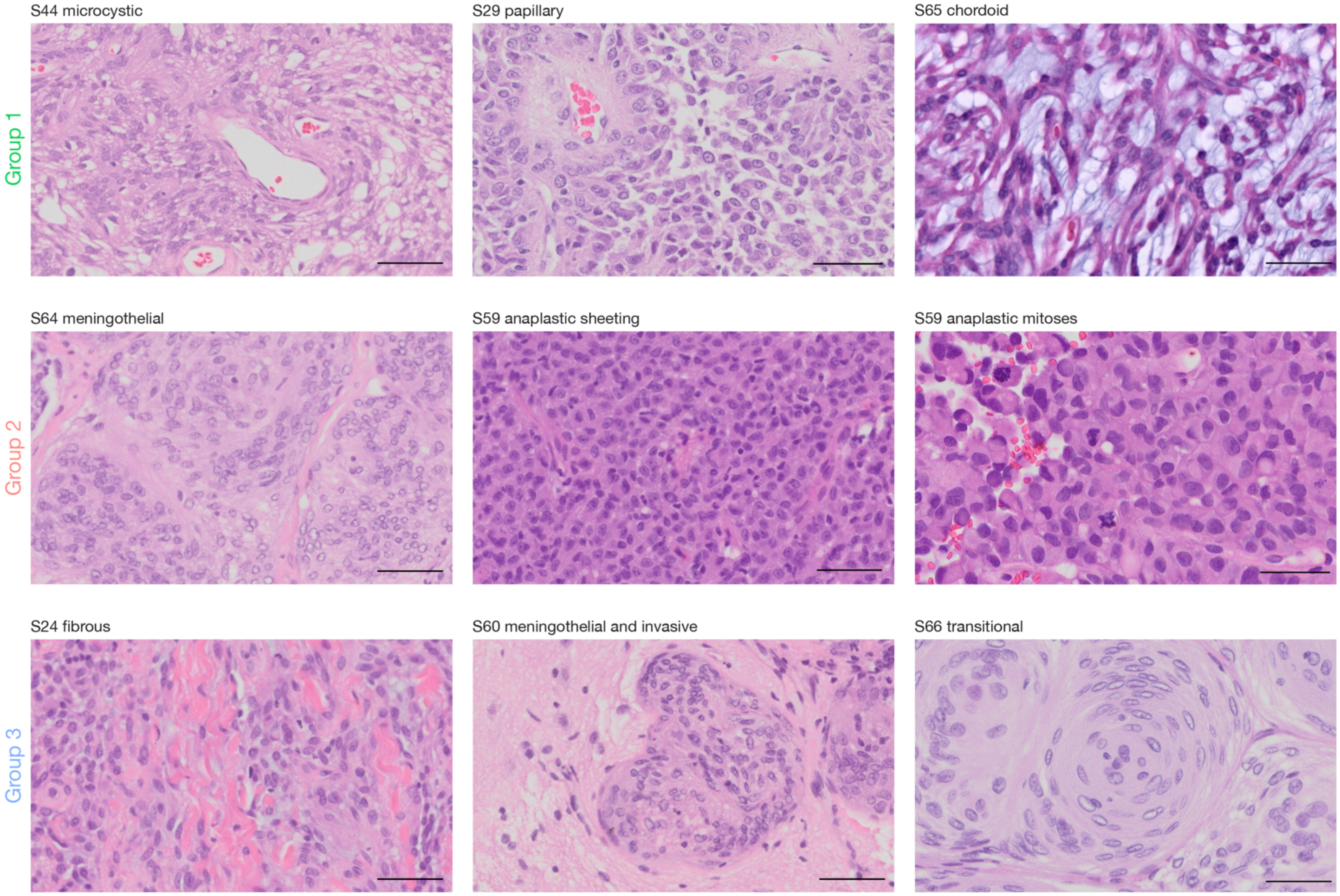

Supplementary Table 1. Canine meningiomas.

Supplementary Table 2. Differentially expressed genes across DNA methylation groups of canine meningiomas.

Methods

Canine meningiomas

Tissue samples from canine meningiomas were obtained from clinical cases presenting to the University of California Davis Veterinary Medical Teaching Hospital. Samples were collected via biopsy or at necropsy within 1 hour of euthanasia and were snap frozen and stored in liquid nitrogen. All pet owners signed a VMTH informed consent form (approved by the University of California Davis Animal Care and Use Committee, #22002). All tumors were histologically classified as meningiomas according to the World Health Organization Classification of Tumours of the Central Nervous System [11].

Nucleic acid extraction

Genomic DNA and total RNA were isolated simultaneously from frozen canine meningiomas using the Allprep DNA/RNA Mini Kit (Qiagen, Cat#80204). Genomic DNA quantity and quality were assessed using a Nanodrop 8000 (ThermoFisher) and a Qubit 4 Fluorometer with the Qubit 1x dsDNA HS Assay Kit (ThermoFisher, Cat# Q33230). All samples processed for whole genome bisulfite sequencing had absorbance ratios A260/280 of 1.8-2.0 and total DNA quantity >200ng. Total RNA quality and quantity were assessed using a Nanodrop 8000 and an Agilent 4200 Tapestation with RNA Screen Tape (ThermoFisher, Cat# 5067-5576) and RNA Screen Tape sample Buffer (ThermoFisher, Cat# 5067-5577). All samples processed for RNA sequencing had RIN >8 and a total RNA quantity >100ng.

Whole genome bisulfide sequencing and analysis

Whole genome bisulfite sequencing (WGBS) samples were pooled and sequenced on 8 NovaSeq 6000 S4 using EZ DNA Methylation-Gold Kit/Accel NGS Methyl-Seq DNA Library preparation and paired-end sequencing. Samples had 513M to 2853M pass filter reads, with Q30 above 87%. Samples were mapped using Dragen and the percent total mapping against reference genome GSD 1.0/canFam4 was 95%. Uniquely mapped reads were above 60%. Library complexity (i.e. the percentage of non-duplicate reads) was determined by measuring the percentage of unique fragments in mapped reads using MarkDuplicate. Percent duplicated reads were between 8% to 34%. Coverage statistics were also measured using Dragen. The mapped sequencing depth coverage (after alignment and marking duplicates) was between 15x to 118x. The mean insert size was between 168 and 220 bases. More than 30% of each genome had coverage above 20x. Methylation calling was performed using Dragen. The mapping efficiency (i.e. number of sequences with a unique best alignment) against the reference genome was 83-88%. There were 42.39-83.64% of cytosines methylated in the CpG context, 0.36-1.91% cytosines methylated in the CHG context, and about 0.35-2.27% cytosines methylated in the CHH context.

For clustering analyses, raw sequencing reads were processed for quality control using FastQC v0.12.1 (https://www.bioinformatics.babraham.ac.uk/projects/fastqc/). Reads were trimmed over two sequential steps using Trim Galore v0.6.10. First, adapter sequences and low-quality bases (Phred score < 30) were removed from both the 5' and 3' ends of all reads. second, ten poly-A bases were further trimmed from the 5' end of all reads. The *Canis lupus familiaris* reference genome (CanFam3.1.104) was prepared for bisulfite mapping using the Bismark v0.24.0 Genome Preparation function, which converts all "C" bases to "T" in the reference genome and subsequently creates an index [8]. Trimmed reads were aligned to the prepared reference genome using Bismark with the --bowtie2 and --non_directional options, which performs bisulfite-aware alignment and automatically extracts methylation data. Aligned reads were processed to remove duplicates using the deduplicate_bismark function. The Bismark Methylation Extractor function was used to extract methylation statuses for the CpG loci from the aligned BAM files. To further process the data for visualization and statistical analyses, bedGraph files were generated from methylation data using the bismark_bedGraph_scaffold function.

CpG context files were preprocessed for clustering analysis using MethylKit v1.26.0 [2] in R v4.3.1. Methylation calls were filtered to those with a minimum coverage of 10 reads per CpG position. CpG sites with coverage exceeding the 99.9^{th} percentile, most likely resulting from PCR bias, were removed. Hierarchical clustering and principal component analysis (PCA) were performed on filtered data using the clusterSamples and PCASamples functions, respectively. CpG methylation percentage was calculated for each sample, and PCA was performed on the resulting matrix. PCA scores and variance explained were exported as CSV files. The prepared data were processed using different clustering methods to examine the inherent groupings within

the data, including (1) hierarchical clustering to identify nested clusters based on Euclidean distance metrics, (2) k-means clustering to partition CpG sites into k distinct clusters based on their features, and (3) PCA for dimensionality reduction before clustering. Plots for visualization were generated using the pheatmap and ggplot2 packages. Heatmaps were generated for all CpG sites as well as for the top 2,000 most differentially methylated CpG sites. UMAP and t-SNE were applied on the scaled matrix of methylation percentages to reduce dimensionality, with results visualized using ggplot2. Consensus clustering was performed using the ConsensusClusterPlus package to validate the stability of the clusters. Tiling window analysis was performed to examine regional methylation patterns across the genome, specifying a window size of 1kb, 2kb, or 4kb bases and a step size of 1kb bases. Clustering and PCA were performed on the resulting regional methylation data.

Hypomethylated regions (HMRs) were identified using MethylSeekR v1.40.0 [3]. CpG methylation data for each sample was read from the Bismark bedGraph file using the read.delim function in the gdata package. Data were filtered to only include bases with a minimum coverage of 5 reads, and genomic feature annotations including promoters, exons, introns, and intergenic regions were acquired from the UCSC Genome Browser. CpG island, shore, and other regions were also obtained from the UCSC Genome Browser to provide a comprehensive annotation landscape.

Copy number variant analysis

Copy number variants (CNVs) were extracted WGBS data and analyzed using CNVnator with default settings [1]. To estimate the percent of the genome affected by CNVs, data were filtered to retain CNVs with p-values less than 0.05, effectively excluding CNVs with poor statistical support. For each sample, the lengths of all CNVs meeting these filtering criteria were summed and divided by the total size of the *Canis lupus familiaris* genome (2,203,764,842 base pairs) to calculate the percentage of the genome affected by CNVs. The calculated percentages were visualized using ggplot2 in R.

RNA sequencing and analysis

Between 100ng and 1ug of total RNA was used as input for RNA sequencing libraries. Libraries were generated using the Illumina TruSeq Stranded mRNA library kit according the to the manufacturer recommended protocol. Libraries were pooled and sequenced on NovaSeq S1 using a 2x150 cycle kit. HiSeq Real Time Analysis software v.3.4.4 was used to process raw data files. Illumina bcl2fastq2.17 was used to demultiplex and convert binary base calls and qualities to fastq format. Samples had 44 to 61 million pass filter reads with more than 91% of bases above the quality score of Q30. Reads were trimmed for adapters and low-quality bases using Cutadapt. Trimmed reads were mapped to the CanFam4 reference genome (GSD_1.0 from NCBI) using STAR aligner v2.7.0f with two-pass alignment. RSEM v1.3.1 was used for gene and transcript quantification based on the CanFam4 GTF file. The average mapping rate of all samples was 83% with unique alignment above 66%. There were 13.13-26.26% unmapped reads. Mapping statistics were calculated using Picard. Samples had between 0.01-0.76% ribosomal bases. Percent coding bases were between 58-71%. Percent UTR bases were 10-16%, and mRNA bases were between 75-82% for all the samples. Library complexity was measured in terms of unique fragments in the mapped reads using Picard MarkDuplicate, revealing 48-78% non-duplicate reads.

For clustering analyses, raw sequencing reads were processed for quality control using FastQC v0.11.9 (https://www.bioinformatics.babraham.ac.uk/projects/fastqc/). Reads were trimmed to remove low-quality bases and adapter sequences using Cutadapt v3.7, with a minimum read length of 20 bases and a quality threshold of 30 [13]. The *Canis lupus familiaris* reference genome (CanFam3.1.104) was indexed using HISAT2 v2.2.0, and trimmed reads were aligned using HISAT2 with default parameters [7]. Read counts were generated at the exon level using featureCounts from the Subread package v2.0.6 specifying exon as the feature type, gene_id as the attribute for grouping, and including gene_name and gene_biotype as extra attributes [10]. For statistical analyses, count data were imported into R v4.3.1 and analyzed using the DESeq2 package v1.40.2 [12]. Genes with less than 10 counts across all samples were removed, and counts were normalized and transformed using variance-stabilizing transformation (VST). For visualization, the following R packages were used: ggplot2 for general graphics, pheatmap for heatmaps, and DESeq2 for PCA plots and other visualizations [12]. Gene ontology analyses were performed in ENRICHR.

For pathway enrichment and visualization, preranked Gene Set Enrichment Analysis (GSEA, v.4.3.2) was performed to identify pathways enriched in differentially expressed genes. The gene rank scores were calculated using SIGN(log2FC) × −log10(p-value). Pathways were delineated by the gene set file Human_GOBP_AllPathways_no_GO_iea_July_03_2023_symbol.gmt, which is periodically updated and maintained by the Bader laboratory. Positive and negative enrichment profiles were achieved through 2000

permutations. The gene set size was constrained between 10 to 500 for the analysis. Pathway analysis results were visualized with the EnrichmentMap App (v.3.3.6) through Cytoscape (v.3.10.0). Parameters set for nodes included an FDR q-value of less than 0.05, a p-value of less than 0.05, and nodes that shared gene overlaps with a Jaccard + Overlap Combined (JOC) threshold of 0.375. Such nodes were interconnected with a blue line (edge) to formulate network maps. Clusters of analogous pathways were pinpointed and labeled using the AutoAnnotate app (v.1.4.1) in Cytoscape. This app incorporates a Markov Cluster algorithm, connecting pathways by mutual keywords in their description, which underwent editing for clarity. The resulting groups of pathways were designated as the major pathways in a circle.
